# Can the Dimensional Optimisation of 3D FDM-Manufactured Parts Be a Solution for a Correct Design?

**DOI:** 10.3390/ma18020408

**Published:** 2025-01-16

**Authors:** Adrian Neacșa, Alin Diniță, Ștefan Virgil Iacob

**Affiliations:** 1 Mechanical and Electrical Faculty, Petroleum-Gas University of Ploiesti, 100680 Ploiesti, Romania; 2 Industrial Engineering and Robotics Faculty, Politehnica University of Bucharest, Spl. Independentei 303, 060042 Bucharest, Romania; 3 Economic Sciences Faculty, Petroleum-Gas University of Ploiesti, 100680 Ploiesti, Romania

**Keywords:** 3D printing, polymers, geometric accuracy, design, compliant products

## Abstract

Additive manufacturing technology, also known as 3D printing, has emerged as a viable alternative in modern manufacturing processes. Unlike traditional manufacturing methods, which often involve complex mechanical operations that can lead to errors and inconsistencies in the final product, additive technology offers a new approach that enables precise layer-by-layer production with improved geometric accuracy, reduced material consumption and increased design flexibility. Geometrical accuracy is a critical issue in industries such as aerospace, automotive, medicine and consumer goods, hence the importance of the following question: can the dimensional optimisation of 3D FDM-manufactured parts be a solution for correct design? This paper presents a complex study of model parts printed from four common polymers used in fused deposition modelling (FDM) additive technology, namely ABS (acrylonitrile–butadiene–styrene), PLA (polylactic acid), HIPS (high-impact polystyrene) and PETG (polyethylene terephthalate glycol). The results of the methodology used highlight the dimensional changes that need to be made at the design stage, depending on the direction of printing and the type of geometric elements in the final part.

## 1. Introduction

Modern industry is facing increasing demands for efficiency and competitiveness in the manufacturing of mechanical parts. In this context, additive technologies have become increasingly important in recent years as a viable solution for improving manufacturing processes.

Additive manufacturing, or 3D printing, has transformed modern manufacturing. One key area where additive technology has shown significant potential is in improving the geometric accuracy of polymer parts [1,2,3,4]. Conventional manufacturing methods often involve multiple, complicated steps, tools and processes that may result in errors and inconsistency in the finished part. In contrast, additive technology offers a novel approach that enables precise manufacturing on a layer-by-layer basis, resulting in improved geometric accuracy, reduced material waste and increased design flexibility [5,6,7].

Geometric accuracy is a critical issue in industries such as aerospace, automotive, medicine and consumer goods. It refers to the degree of conformity between a manufactured part and its intended design specifications. Achieving high geometric accuracy ensures that parts fit perfectly, perform as intended, meet strict quality standards and a number of studies have accounted for production deviations of the reference model from the designed one [8,9]. Traditional manufacturing methods, such as injection moulding and CNC machining, have limitations when it comes to producing intricate and complex geometries, often requiring costly and time-consuming iterations to achieve the desired level of accuracy.

Additive technology represents a paradigm shift in that it enables parts with complex geometries to be produced directly from a digital design. The process starts with the creation of a 3D model by means of computer-aided design (CAD) software or, in some more complex cases, creating a data-driven system to optimise the additive manufacturing process according to specific constraints [10]. The digital model, which serves as a roadmap for the additive manufacturing equipment, is then sliced into thin layers. The technology works by adding layer upon layer of material, often in the form of resins or polymers, which are selectively hardened or melted. Obviously, a number of parameters must be taken into account for the creation of parts using this technology, such as the deposition direction, which influences the mass distribution of the material [11], the solidity ratio [12,13], the yield strength [14] and many others.

The ability to produce complex shapes with high precision is one of the key benefits of additive manufacturing [15,16]. Traditional subtractive manufacturing is constrained by tooling limitations and may require multiple set-ups to achieve the desired form, resulting in accumulated inaccuracy. Additive technology, on the other hand, enables the creation of intricate and organic designs without the constraints of traditional tooling. This is particularly beneficial as additive manufacturing can optimise designs for both structural integrity [17] and weight reduction in industries that require lightweight yet strong components.

Additive manufacturing also makes it possible to create internal structures which are otherwise impossible or extremely difficult to achieve using conventional methods. These structures, often referred to as lattice or cellular structures, can enhance the mechanical properties of polymer parts [18]. Manufacturers can reduce overall material usage while maintaining or improving strength-to-weight ratios by strategically placing voids within a part. This not only contributes to greater geometric accuracy, but also to the conservation of resources and the sustainability of the manufacturing process [19].

Realising the full potential of additive manufacturing to improve the geometric accuracy of polymer parts, however, remains challenging. Factors such as material selection, process parameters and post-processing techniques have a significant impact on the final accuracy of parts. Material properties such as viscosity, thermal expansion and cure behaviour can lead to variations in part dimensions during the additive manufacturing process. Additionally, inadequate cooling mechanisms, or uneven thermal distribution, can cause parts to warp and distort, affecting their overall accuracy and long-term dimensional stability, which may be affected by changes in temperature and humidity or exposure to ultraviolet radiation [20].

Process parameters such as layer thickness, print speed and nozzle size need to be precisely controlled to ensure that the geometry is consistent. Any deviation from the optimum parameters can introduce errors that can lead to inaccuracies in the final part [21]. In addition, challenges related to build orientation and support structures can affect accuracy. Certain geometries may require additional support during printing to prevent sagging or deformation. Removing this support may result in surface irregularities and dimensional changes.

To achieve the desired geometric accuracy, considering post-processing threats is also essential. Additively manufactured parts often require cleaning, curing and sometimes machining to refine surfaces and dimensions. Poor finishing can offset the benefits of additive manufacturing by introducing errors or inconsistencies that compromise geometric accuracy. Therefore, the development of reliable and efficient post-processing techniques is essential if the full potential of additive technology is to be realised.

Additive technology has revolutionised manufacturing by providing unprecedented opportunities to improve the geometric accuracy of polymer parts. Through layer-by-layer manufacturing, complex geometries can be achieved with precision and efficiency. This leads to reduced material waste [22] increased design freedom and improved mechanical properties. While challenges remain in terms of material behaviour, process parameters and post-processing, ongoing research and technological advances are continually addressing these issues [23].

The impact of additive technology on geometric accuracy transcends traditional industry boundaries, opening avenues for innovation in product design, healthcare and beyond. As materials and processes evolve, additive manufacturing is poised to further modernise manufacturing by pushing the boundaries of what is achievable in terms of geometric accuracy and design complexity. With continued interdisciplinary collaboration and advances in materials science and engineering, the potential for additive technology to transform the manufacturing landscape appears limitless, offering a future where precision and complexity converge in harmony.

The stages of this article are summarised in a graphical abstract based on the logic of the initiation and conduct of the study.

## 2. Literature Review

In order to highlight the research that has been undertaken in this area of engineering, a keyword-based, in-depth review of some relevant articles exploring the complex relationship between polymer materials, geometric features and FDM additive technology is provided. It covers a wide range of critical issues, from process parameter and material selection to geometric optimisation, mechanical behaviour, surface finish, tolerances and innovative applications in diverse fields and sustainability. The synthesis of these articles provides a valuable insight into both the challenges and opportunities that FDM presents for creating complex, customised and sustainable geometries using different polymer materials.

Budzik et al. [24] introduced a quality control approach tailored to polymer additive manufacturing products, with the specific methodology varying according to the application. Designs are divided into two groups: those for visual presentation and those directly supporting the manufacturing process. In addition, the authors propose a comprehensive control system that is in line with the requirements of Industry 4.0. There are three stages in the quality control process, data control, manufacturing control and post-processing control, based on the use of models. Various materials were used in the research, such as RGD 720 photopolymer resin (PolyJet method), ABS M30 thermoplastic resin (FDM method), E-partial photopolymer resin (DLP method), PLA thermoplastic resin (FFF method) and ABS thermoplastic resin (MEM method). The accuracy of the measurement equipment used was significantly higher than the accuracy of the production technologies used. The results show that the PolyJet method is the most accurate, while the MEM method is considered to be the least accurate. In addition, it highlights the importance of considering not only model specificity and purpose, but also economic factors in the selection of materials, 3D printing techniques and measurement methods, as not all products require high levels of accuracy and durability.

In recent years, there has been a significant focus on additive technology. This is largely due to its remarkable ability to produce intricate shapes and its suitability for creating customised products. Recognising the pivotal role of additive manufacturing in shaping the future of manufacturing, product design, process development, material selection and many other aspects have been comprehensively studied [25]. Particularly in design methodologies, a variety of design approaches have been proposed specifically for additive manufacturing. Several computer-assisted programmes have been developed, including those based on geometric modelling, bio-inspired design, evolutionary algorithms and topological optimisation. However, the authors have identified a potential problem in the way these methods are often referred to as ‘design for additive manufacturing’ without specification of the specific additive manufacturing process. This lack of specificity could prove challenging, as a generalised design approach may not match the best practices for a particular additive manufacturing process. Therefore, a thorough literature review focusing on design methodologies for 3D polymer printing using material extrusion is the main objective of this paper. While highlighting areas of research that are still underdeveloped, the paper aims to provide a comprehensive overview of the current state of the field.

The creation of complex components based on computer-designed designs is promised using 3D printing for tooling and rapid manufacturing. However, the inherent limitations in the mechanical properties and functionality of pure polymer print parts necessitate the development of higher-performance printable polymer composites. Three-dimensional printing is an attractive method for producing composites due to its advantages such as precision, cost-effectiveness and customisable shapes. Wang X. et al. [26] reviews 3D printing techniques for polymer composites, examines the properties and performance of resulting printed composite parts and explores potential biomedical, electronics and aerospace applications. It presents common 3D printing methods including fused deposition modelling, selective laser sintering, inkjet 3D printing, stereolithography and 3D scanning. The formation process and the performance of composite materials reinforced with particles, fibres and nanomaterials will be highlighted. Finally, as a catalyst for future research in 3D printing, the main limitations are identified.

Kermavnar T. et al. [27] investigated the use of additive manufacturing (AM) in developing ergonomically designed products, focusing on customisation and iterative design. To examine methodological aspects of studies in this area, they conducted a systematic literature review. The search included terms like ‘3D printing’*, ‘additive manufacturing’* and ‘human factors’. Inclusion criteria included studies that described in detail the use of AM for the ergonomic design of products/prototypes, together with a comprehensive description of the ergonomic testing methodology used. A total of forty studies were identified from a variety of fields, including medicine, assistive technology, wearable technology, hand tools and test equipment. The predominant technology used was fused deposition moulding using polylactic acid. However, acrylonitrile–butadiene–styrene emerged as the preferred material. Various methods were used to evaluate the products, including objective, subjective, qualitative and quantitative. Recommendations to guide the selection of the most appropriate AM technologies and materials for specific ergonomic applications were made on the basis of these findings.

Mwema F.M. et al. [28] also demonstrate the use of dimensional analysis and microscopy to assess the quality of home-made 3D-printed parts. Using an inexpensive fused deposition modelling (FDM) 3D printer, they produced several common geometric shapes using PLA filament. These included circles, diamonds, hollow shapes, squares and S-shapes. These designs were first created as 3D computer-aided design (CAD) models, and the G-codes required for printing were generated using CURA’s slicing software. After the printing process was completed, they carefully measured the dimensions of the printed components and then compared these measurements to the specifications in the CAD models. Their analysis revealed notable discrepancies in certain areas, particularly the tips of the diamond shapes, the corners of the shapes and the thickness of the S shapes. Optical microscopy was then used to investigate the causes of this variation. Their microscopic analysis revealed that the main cause of the dimensional variation was insufficient fusion of the filament material during the printing process. This finding, particularly in areas where fusion needs to be improved, provides valuable information for improving the quality and precision of the 3D printing process.

Additive manufacturing (AM) or 3D printing offers several key advantages, including design flexibility, personalised mass production, waste reduction, the capability to create intricate structures, and rapid prototyping. This review thoroughly covers the main 3D printing techniques, materials, and their cutting-edge applications. It specifically highlights significant breakthroughs in the use of AM in diverse fields such as biomedical, aerospace, construction, and protective structures. The current state of materials development in 3D printing is explored, encompassing metal alloys, polymer composites, ceramics, and concrete. Ngo D.T. et al. [29] also addresses the essential challenges in AM processing, including issues like void formation, anisotropic behaviour, computational design limitations, and the noticeable layered appearance of printed objects. In summary, this paper serves as an all-encompassing overview of 3D printing, presenting both its advantages and disadvantages, rendering it a valuable reference for future research and development endeavours.

It is essential to understand the variations in both geometric and mechanical properties of components to effectively design and optimise them for additive manufacturing (AM) processes. Mueller J. et al. [30] focus on a systematic investigation of these variations in 3D-printed structures. The first objective is to analyse the various parameters that influence the process as a whole. The aim is to identify and quantify the parameters that lead to the most precise geometry and superior mechanical properties. Once this comprehensive understanding has been gained, it will be possible to produce accurate models of the components and then to optimise the manufacturing process. Successful manufacture and testing of the components follow from this optimisation. The research identifies several significant factors that affect mechanical properties, ranked in decreasing order of influence: the number of intersections between layers and nozzles in the direction perpendicular to the load, the exposure time to ultraviolet light during printing, the position of the structure on the printing table and the expiration date of the raw material used. Problems such as nozzle blockage have a significant effect on the geometry of the printed structures, and the warm-up time of the machine also plays an important role. It is worth noting that surface roughness is not affected by any of these factors, although storage time plays a minor role. Given the rapidly changing nature of the AM materials landscape, it is important to recognise that the characterisation process will need to be iterative. The research demonstrates how the Design of Experiments (DOE) method can be effectively used to create a cost- and time-efficient design with a high degree of statistical accuracy. This allows researchers and manufacturers to remain agile and adapt to evolving materials and conditions.

For prototyping and low-volume production of functional parts, additive manufacturing is a valuable and accessible tool. Commonly used materials include polylactic acid (PLA) and thermoplastic polyurethane (TPU). The characterisation of 3D-printed PLA and TPU is essential for effective design and finite element modelling of functional parts. Taking into account design parameters such as size and filler content, Elmrabet N. et al. [31] investigates the mechanical properties of additively manufactured PLA/TPU samples. PLA/TPU 3D-printed parts were produced using ISO standard geometries with 20%, 60% and 100% infill. Tensile and compression test results indicate that conventional ISO test standards may not fully characterise 3D-printed materials for finite element modelling or practical applications. The interplay between infill and build size significantly affects the mechanical performance of 3D-printed parts. Varying the size of the part can cause mechanical properties to differ between large and small sections of the same part. For small cross-section parts, where reducing the nominal infill had a milder effect on the resulting samples, this effect was less pronounced. The results suggest that material properties may not be homogeneous in 3D-printed functional parts with significant dimensional differences between sections [32]. This is an important consideration for designers who are using 3D printing in applications that involve mechanical loading.

Several studies have focused on cellular metamaterials inspired by nature, leading to global innovation. However, these studies have primarily used single materials with limited multifunctionality. The advent of additive manufacturing (AM) has revolutionised the creation of complex structures by incorporating multiple materials, resulting in improved capabilities, adaptability to various environments, and enhanced mechanical properties. Recent endeavours have delved into diverse materials, methodologies, design approaches and optimisation techniques for multi-material additive manufacturing (MMAM). Despite several years passing, comprehensive reviews in this field have been scarce. Nazir A. et al. [33] provide an in-depth review of MMAM systems, emphasising the fundamental principles of their core processes. The review systematically covers material combinations, along with associated design, modelling and analysis strategies. The authors also highlight cross-industry applications, opportunities and post-processing considerations for MMAM-produced components. They identify limitations and challenges in existing software tools, MMAM processes, materials, and interfaces between different materials. The paper concludes by exploring potential strategies to overcome these technological barriers, outlining future directions in this domain. This comprehensive perspective serves as a valuable resource for researchers and engineers engaged in the design and fabrication of intricate, nature-inspired objects.

Xiangren Kong et al. [34] made an experimental study on the failure behaviour of the interface of 3D-printed parts made of fibre-reinforced composite materials. Thus, they observed the internal quality of the pure Onyx specimen print by noting that the voids are regularly presented, the porosity is about 16% and the shape of the voids is somewhat uniform. They also studied the internal print quality when continuous carbon fibre and Onyx are cross-laminated, finding that the continuous layer of carbon fibre appears to form a whole body and there is no major gap between adjacent filaments in each layer. This is because the print head will press down on the fibre to ensure that the fibre continues to properly adhere to the underlying layer. After the study, they highlighted the fact that the fusion effect of a single type of printed material (i.e., pure Onyx or pure continuous fibre) is quite good; on the other hand, for printing different materials, there could be an obvious separation at the interface of the two materials, implying that fusion at the interface may not be ideal.

Zapciu A. and Constantin G. [35] highlight the problems related to the thermal stresses inside the part in the case of 3D printing with polymers at high temperatures in the ambient environment and propose a method to improve the technological process using a camera with vacuum. As a result of the tests, it was found that the parts made of polyetherimide (PEI—ULTEM 1010) acquired a 14% increase in strength when it was printed in an environment using a vacuum chamber, and the parts made under the same conditions from acrylonitrile–styrene–acrylate (ASA) did not show significant differences.

Wang X. et al. [36] focused on the effects of porosity on the mechanical properties of 3D-printed polymers, looking at the three-dimensional microscopic details of the internal pores, such as size, shape, density and spatial position, which were quantitatively evaluated by X-ray computed tomography (XCT), subsequently conducting experiments to highlight the mechanical properties of the obtained materials.

Another study involved analysing the tribological qualities of 3D-printed PLA polymers and whether they are affected by the existence of different colours. In this sense, Muammel M. Hanon and Laszlo Zsidai [37] used the technology of fused filament deposition modelling (FDM) for the tribological production of samples. The impact of print orientation and colour was studied by producing samples in different directions (horizontal, 45-degree angle and vertical) and different filament colours (white, black and grey), while performing tribological tests alternately, applying two different loads (150 and 200 N). Attention was also paid to surface roughness and product hardness, as these are important aspects for understanding the tribological behaviour. As a result of the study, it was found that with regard to the colour of the filament, it has an obvious impact on the friction coefficient test of the parts under all conditions. Thus, white samples have the highest coefficient of friction, while the lowest was observed in grey, and black samples showed a high rate of wear depth. All samples under low load showed a significant bond slip tendency compared to high load. The 450 vertical parts showed deformations in the contact area, and the wear rate of the 450 parts was high at low load printing, showing larger gaps between the printed layers. Vertically printed samples showed abrasive wear confirmed by scar marks, pits and grooves due to deep asperities.

Some research has looked at the influence that the size and orientation of the 3D-printed fibre have on the mechanical properties of the product. Thus, Juracka D. et al. [38] placed the fibres along the longitudinal axis and perpendicular to the axis. Five samples were made on a given fibre direction for each set, so a total of twenty samples were tested for each category (N 0.4 and N 0.8). It was found that, for orientation along the longitudinal axis, no failure of the sample was indicated during the test when there was only a gradual increase in deformation, and in the case of orientation perpendicular to the axis, the samples broke before the resistance limit was reached.

Another study involved testing the surface durability of 3D-printed gears using PLA, Tough-PLA and TPU materials. Chenxiao Li and Chul-Hee Lee [39] started from the premise that, by combining two materials with different characteristics, it is possible to improve the mechanical properties of gears. For testing, a series of parameters such as gear speed and working temperature were taken into account, and later, the uses were analysed using scanning electron microscopy (SEM). It was found that, among PLA, TPU and Tough-PLA gears, the combined Tough-PLA gear has the best surface wear resistance. Also, the improvement of transmission efficiency is given using TPU, a flexible material that is better for this mechanical system which generates vibrations during operation which lead to energy loss. By reducing the vibration of the gear through the material used, the wear of the gear surface is reduced. Also, by combining the two materials, the surface part of the tooth is made of a material with higher hardness and thus the wear resistance is improved.

Another study involved the use of FDM (multi-material additive manufacturing) technology with a single extruder. Practically, Mohammad Rafiee et al. [40] points out that multiple materials in any available form can be co-fed into a single-screw extruder and subsequently deposited on the print bed. This technology may have the ability to print a structure with controllable and variable compositions. The use of multi-material 3D and 4D printers increase the improvement of the quality of parts by changing the composition or type of material, which cannot be easily achieved using classical technologies. The main characteristic of multi-material 4D printing is the geometric transformation after 3D printing through shape changes (memory effect). In other words, you can cause the 3D-printed part to shrink, expand or fold, which is actually the fourth dimension. Shape memory polymers (SMPs) and shape memory alloys (SMAs) are two different types of materials that are used for 4D printing.

An interesting study involved analysing the strain rate sensitivity of five different thermoplastic polymers obtained by melt filament manufacturing: polylactic acid (PLA), acrylonitrile–butadiene–styrene (ABS), polyethylene terephthalate glycol (PETG), polyamide (PA6) and polypropylene (PP). Following their, study Vidakis N. et al. [41] found that there is a difference in tensile strength according to different elongation rates. The printed samples (ABS, PA6, PETG, PLA and PP) show a low dependence on mechanical deformation (stiffness, flow resistance, tensile strength and toughness). However, it was found that PLA, compared to the other materials, seems to be more sensitive to the strain rate.

Another study involved the printing of multi-material parts that have soft and rigid materials (PLA and TPU) in their structure. Guo Liang Goh et al. [42] conducted a detailed examination of interlaminar adhesion by manufacturing and testing three types of test specimens, differentiated by interface treatments: unmodified specimens; samples where an interlocking mechanical design was introduced, featuring two layers of interlocking geometry that extends up to 2 mm into the material; samples that have been altered by modifying the upper filling, so as to increase the surface bond. The goal was to enhance the mechanical connection between the layers through physical programming. It was found that the filler changes resulted in superior interlaminar bond strength, thus achieving a 25% improvement over the standard sample. These significant results can be useful in the design and fabrication of multi-material components, as interlayer adhesion is critical.

Commonly known as FDM™ (fused deposition modelling), FFF has become widely recognised as a versatile, reliable and cost-effective additive manufacturing (AM) technique. Initially adopted by industry for rapid prototyping, FFF has more recently been adopted by the general public. Despite significant advances in printer technology and filament materials, producing robust, high-performance parts for demanding applications remains challenging. Inherent problems such as voids and poor interlayer adhesion mean that FFF-printed parts have inferior mechanical properties compared to conventionally manufactured counterparts. This is particularly relevant given the growing demand for customizable porous structures in areas such as biomedicine, 4D printing and lightweight cellular composites. It has become essential to understand the challenges posed by pores. This review focuses on recent findings in void formation, categorisation, research methods and mechanisms, while previous research has addressed the importance of interlayer bonding. The primary aim is to comprehensively explain the two main current approaches to studying voids: quantitative analysis and imaging. The influence of feedstock and printing parameters on void formation is also discussed in detail. Finally, this review identifies gaps in existing research and highlights unexplored challenges related to void formation and how it affects the mechanical performance of FFF parts.

Based on this brief literature review, the authors identified the main research gaps in the area of the influence of print direction and type of polymer material on the dimensional accuracy of different geometric elements of parts produced using additive FDM technology.

In this way, the importance of this article has been established in terms of the geometric accuracy of parts made by additive technology from four different polymeric materials. This has implications for the correspondence between the dimensions imposed by the 3D CAD model and those of the final parts, so that they do not end up as technological scrap.

## 3. Materials and Methods

As presented in the introductory chapter, this study is based on the measurement of the dimensions of three-dimensional models printed using FDM additive technology from four types of materials, namely: PLA, ABS, HIPS and PETG. The materials, the printer and the complex measuring device used are described in the following subsections.

### 3.1. Materials Used

#### 3.1.1. ABS (Acrylonitrile—Butadiene—Styrene)

ABS (acrylonitrile—butadiene—styrene) is a thermoplastic that is durable, tough and easy to work with. The material has a long history of use in various industries, and more recently in 3D printing. ABS can be used in a wide range of applications, including materials for consumer goods and housings for household appliances and toys. ABS is a material that is rigid, strong and impact resistant. It is resistant to high temperatures, impact and abrasion. It is highly resistant to chemicals and ultraviolet rays. It is a plastic that can be finished by grinding or painting.

Expanding ABS in 3D printing occurs when the ABS material heats up and expands during the printing process. This can cause problems, especially at high printing temperatures or for large and complex parts, such as warping or cracking of the printed parts.

To prevent ABS from expanding, it is important to consider the printing temperature of the material and to use a suitable printing platform, such as a heated platform. It may also be useful to use racks to cool parts during printing. Conversely, ABS shrinks as it cools and shrinks, which can cause distortion and gaps in printed parts. This can be more pronounced in parts that are large or complex in shape or when low print temperatures are used.

In order to prevent the shrinkage of ABS, it can be useful to use cooling systems to cool down the printed parts quickly after the printing process has been completed [43]. Adjusting the print temperature or using a less shrink-sensitive material, such as PLA, can also help.

The principal factors that can cause dimensional differences in measurements include:

- Thermal expansion of material (ABS) during 3D printing. The melting temperature of ABS is approximately 220–250 °C and, during the cooling process, the temperature can vary depending on the environment, which can result in the shrinkage or enlargement of the printed object.
- Failure to calibrate the 3D printer. If the machine is not calibrated correctly or if there are system errors in the movement of the axes, this can lead to differences in the size of the printed object.
- Cooling time of the object. After printing, the object should be allowed to cool naturally to stabilise the material. Distortion and inaccurate dimensions can result if the object is removed from the printer prematurely or is not cooled properly.
- Mechanical stress. During the printing process, there are a number of forces that affect the movement of the spindles, such as vibrations caused by the print motors, which can cause the final dimensions of the object to vary.

#### 3.1.2. PLA (Polylactic Acid)

PLA (polylactic acid) is a biodegradable material made from lactic acid [43]. It is produced by fermenting corn or other plants with a high sugar content. It is a popular material in the 3D printing industry because it is easy to work with and has a number of beneficial properties:

- Biodegradability. PLA is a biodegradable material, which means that it will break down in a natural way in a composting environment. This makes it a popular choice for items that do not have to be durable or environmentally sound.
- Ease of processing. PLA is a relatively easy material to work with and process. It can be printed at lower temperatures than other materials. It does not require a heated platform to print successfully. In addition, it is a non-toxic material and does not give off any strong odours when in use.
- A high-quality surface finish. Compared to other materials, PLA 3D-printed objects offer a superior surface quality and finish. They can be machined, painted or varnished to give them a high-gloss appearance.
- 3D printing. During the printing process, the ideal temperature for PLA is around 190–220 °C, but this can vary depending on the specifications of the printer and the material manufacturer. A minimum of 30 mm/s is required for 3D printing with PLA.

The expansion of PLA in 3D printing occurs when the PLA material is heated, which causes it to expand during the printing process. Expansion can cause the cracking or distortion of the printed component, especially at high printing temperatures or for large and complex parts.

It is important to consider the printing temperature of the material and use a suitable printing platform, such as a heated platform, to prevent PLA from expanding. It may also be useful to use a supporting structure to reduce the stress placed on the printed part. However, PLA shrinks when cooling, which may cause warping and gaps in the print. This can be more pronounced in large or complex parts, or when low print temperatures are used.

Optimising the post-print cooling process by allowing the part to cool gradually rather than subjecting it to large temperature swings can help prevent PLA shrinkage. Higher printing temperatures and substrates can also be used. This reduces stress and heat dissipation during printing.

A number of factors can cause dimensional differences in measurements, including:

- 3D printer calibration errors. These can lead to dimensional errors of 2–3%, usually in terms of oversizing the final size.
- Environmental conditions. Even small fluctuations in temperature and humidity can have an effect on the dimensions of the final object. A rise or fall in temperature in the production environment can influence the volume of the material, which in turn can have an effect on the dimensions.
- Material properties. Materials can be affected by changes in ambient humidity and behave differently when heated and cooled, even under the same production conditions. PLA can shrink slightly as it cools, which can cause measuring differences.
- Design accuracy and substrate removal. In the design process, the object may be designed with slightly different dimensions in each direction. This may require accuracy in the substrate’s removal from the finished object.

#### 3.1.3. HIPS (High-Impact Polystyrene)

HIPS (high-impact polystyrene) is a durable plastic. It is mainly used in the packaging industry and for prototyping [43]. This material is similar to ABS, but HIPS is lighter in weight and more flexible, as well as being a more cost-effective choice.

HIPS is often used as a support material for 3D printing, as it can be held in place during the printing process and can be easily removed after the printing process is complete. Although HIPS is a soft thermoplastic polymer, it is lightweight and durable. It is lighter in weight than ABS and is available in a wider range of colours. HIPS has good impact strength and is resistant to both heat and chemicals. It is easy to print HIPS on a 3D printer, although there are a few important things to keep in mind. An extrusion temperature of around 220–240 °C and a heated bed temperature of around 80–100 °C are recommended for printing HIPS.

The expansion of HIPS can occur, especially at high temperatures. To reduce this, it is important to control the print temperature and use a heated print bed to prevent the warping or tearing of the parts.

HIPS can shrink less than other materials such as ABS, but it can shrink, nonetheless. Optimising the cooling process after priming, allowing the part to cool gradually and avoiding large temperature swings is important to minimise shrinkage.

Generally, HIPS is a standard material for 3D printing, and its expansion and shrinkage are important aspects to consider for obtaining accurate and well-machined parts.

A few factors can cause dimensional differences in measurements, including:

- Thermal expansion of high-integrity polystyrene (HIPS) during 3D printing. HIPS has a melting temperature of around 230 °C. During the printing process, the temperature of the environment can vary. This can cause the printed object to shrink or expand. In addition, differences in the temperature of the print bed and the extruder can also lead to differences in the final size of the objects.
- 3D printer calibration errors. Differences in the size of the printed object can occur if the printer is not calibrated correctly or if there are system errors in the movement of the axes.
- Poor adhesion to the print bed. If the adhesion to the print bed is not strong enough, the object may fall out during printing, causing deformation and varying the final size of the printed object.
- Influence of object orientation. The accuracy of the final size of the object may also be affected by the orientation of the object and the direction of extrusion of the material during the printing process.
- Speed of movement of the axes. The final size of the object may be affected by the speed at which the spindles move during the printing process.

#### 3.1.4. PETG (Polyethylene Terephthalate Glycol)

Considered an intermediate between PLA and ABS, PETG (polyethylene terephthalate glycol) is a thermoplastic [43]. It is one of the most popular filament types for 3D printing and has excellent properties, including high strength, flexibility, transparency and long-term durability.

- Strength and durability. PETG is a very tough material and works well in a variety of applications. It has similar durability to ABS and is three times stronger than PLA. It has the ability to withstand impact, which makes it an excellent option for printing parts that need to be able to withstand wear and tear and damage. It also has excellent transparency, which makes it look and feel like glass. As a result, it is a popular choice for the printing of transparent items, such as beverage bottles or other objects.
- Printing with 3D printers. PETG does not require a heated platform surface, but it is recommended to use it at 60–70 °C to improve adhesion to the platform. The ideal temperature for printing with PETG is around 220–250 °C, but this can vary depending on the material manufacturer and printer specifications. This material can print at higher speeds than PLA or ABS, from 50 mm/s, but it is important to adjust print settings to ensure successful printing.

PETG is a popular plastic in 3D printing, known for its strength and durability, and its properties mean that PETG can expand and contract less during printing than other materials, such as PLA.

PETG expansion can be especially noticeable when printing large or complex parts. While PETG shrinks less than other materials, it is important to optimise the cooling process after printing, allowing the part to cool slowly and not exposing it to large temperature fluctuations.

These factors can cause dimensional differences in measurements, including:

- Tolerances in 3D printer precision. Three-dimensional printers have a certain amount of tolerance when processing the shape of objects. This can lead to variations in dimensions. These tolerances, which can be affected by printer set-up and calibration, can be different in each direction.
- Environmental conditions. External factors such as ambient temperature and humidity can affect 3D printing. These conditions can cause the actual dimensions to vary from those designed and can affect the printing process.
- Material used. PETG material has certain shrinkage characteristics that can have an effect on the dimensions of 3D-printed objects. These can be affected by the temperature, the humidity and the density of the material being used.
- Print settings. The final dimensions of the object can be affected by 3D printer settings, such as print speed and layer thickness. These settings can be adjusted so that size variations are a minimum.

A summary of the technical parameters of the materials is given in Table 1.

### 3.2. Three-Dimensional Printer

**Figure 1:**
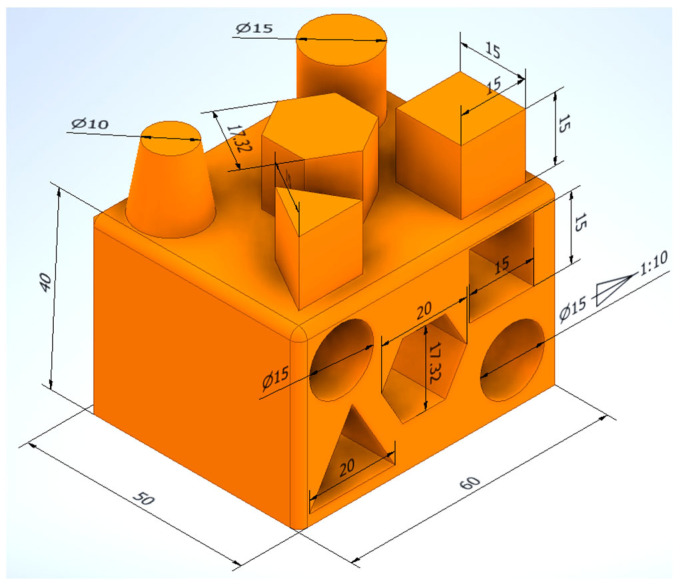
Three-dimensional model part. Source: authors, based on research stage.

All 16 parts in this study have been produced with professional Raise E2 3D printers, which are very well-built and designed for ease of use, resulting in a better 3D printing experience for every application and user. To examine the geometric accuracy of the parts, made by additive technology from PLA, ABS, HIPS and PETG materials, a reference model was first made using specialised CAD software in the educational version. The 3D model of the reference part is shown in Figure 1.

#### 3.2.1. Three-Dimensional Printer Raise E2

The Raise E2 is a desktop 3D printer with independent double extrusion (also known as IDEX). IDEX makes the Raise E2 ideal for professional 3D printing by giving it the ability to perform advanced functions, such as mirror mode and duplication mode. For added convenience when performing maintenance or routine printing tasks, this IDEX 3D printer features easy swap print heads. Easy-to-use features that help produce high-quality 3D-printed parts include the E2’s auto-bed levelling and BuildTak FlexPlate. This desktop 3D printer can print with multiple filament types, and the unique extruder gear design makes the use of flexible 3D printing materials like TPU more reliable and better performing. As with all Raise3D 3D printers, the E2 can be paired with ideaMaker^®^ v5.1.4, a 3D slicing software, the ideaMaker Library, a platform for sharing 3D print files with others in the Raise3D community, and RaiseCloud, a cloud-based 3D printing management platform.

The printer is compatible with all four types of material from which the sixteen 100% infill samples with complex geometric configurations, whose dimensions were to be measured, were made and had been equipped with a print nozzle with a diameter of 0.4 mm.

**Figure 2:**
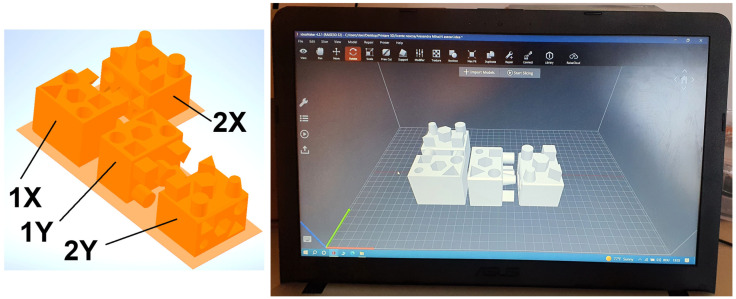
Printing orientation. Source: authors, based on research stage.

The sixteen 100% filled test pieces with different print orientations are shown in Figure 2, and were produced using the optimum print parameters recommended by the manufacturers for each material, as shown in Table 2, in order to evaluate the influence of print speed and nozzle temperature on the geometric accuracy of FDM parts.

#### 3.2.2. Three-Dimensional Filament

Four types of filaments were purchased to make the sixteen pieces, which were printed in different orientations of the extruder feed, using different high-quality materials that were certified by the manufacturers:

- Filament ADURA (ABS) Lucent Orange, 500 g, 1.75 mm diameter;
- Filament UltraFuse (PLA) White, 1000 g, 1.75 mm diameter;
- Filament EasyFil^TM^ (HIPS) Dark Blue, 750 g, 1.75 mm diameter;
- Filament PolyLite PETG Polymaker Teal Turquoise, 1000 g, 1.75 mm diameter.

During the FDM process, the manufacturer’s guidelines for optimal printing parameters were also strictly followed and applied.

### 3.3. Axiom Too HS CMM—Coordinate Measuring Machine

Since 2004, the Axiom Too CMM has been providing the manufacturing industry with a fast and accurate solution to their measurement problems. The Axiom Too HS is an improvement on the standard model. It is both faster and more accurate, without compromising the fantastic value for money for which Aberlink CMMs are renowned.

#### 3.3.1. Axiom Too HS

The Axiom Too HS uses rod drive technology developed for larger machines and vision products rather than a belt drive system. This allows even higher accelerations to be achieved, meaning that the HS model measures approximately 20% faster than the standard model, making it ideal for measuring large volume parts.

Axiom Too HS also uses 0.1 µm resolution scales. Combined with state-of-the-art error mapping techniques, the HS model is extremely accurate, and ideal for close tolerance measurement.

#### 3.3.2. Probe/Head—PH10T (w/TP20, TP200)

The machine on which the Axiom Too HS measurements were carried out is equipped with a PH10T probe and an RTP20 magnetic probe, which uses a volumetric accuracy according to the formula (2.1 + L/250) µm.

The PH10T is a fully motorised probe head offering instant indexing from 0° to 105° in the A axis and to 360° in the B axis in 7.5° increments. Customers who require frequent indexing or more precise alignment to the features to be measured should use this probe.

### 3.4. Method for Dimensional Study of FDM-Printed Parts

**Figure 3:**
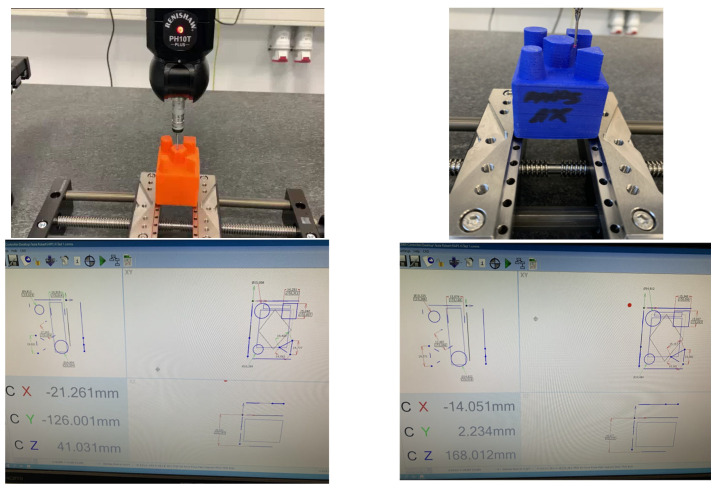
Sample measured with the high-performance Aberlink Axiom Too HS CMM (Vatch Lane, Eastcombe, UK). Source: authors, based on research stage.

In this section, a brief description of the methodology used in this study is provided. Using a Raise 3D printer model E2, a number of sixteen samples, four of each specified material (ABS, PLA, HIPS, PETG), were produced with optimal parameters specified by each material producer and different printing orientations, and their geometric elements were measured using a high-performance Aberlink Axiom Too HS CMM (see the example presented in Figure 3) and the results were recorded in the table format. The data obtained make it possible to determine and analyse the dimensional deviations of the samples from the desired ones in the computer-aided design phase, both in terms of value and in percentage form.

## 4. Results and Analysis

In view of the presentation in the previous section of the materials used, ABS (acrylonitrile–butadiene–styrene), PLA (polylactic acid), HIPS (high-impact polystyrene) and PETG (polyethylene terephthalate glycol), the 3D printer and the complex equipment used to measure the specific dimensions of the geometric elements of the sixteen 3D models printed using additive FDM technology, the results are presented below in both tabular and graphical form.

**Figure 4:**
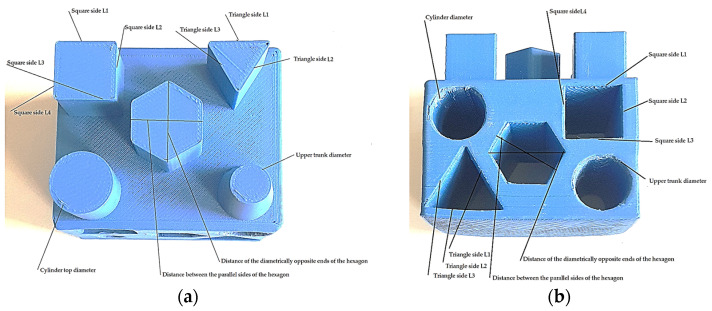
External and internal geometric elements that have been measured by Axiom Too HS CMM. Source: authors, based on research stage. (**a**) External geometric elements; (**b**) internal geometric elements.

The measurement results are in accordance with the methods briefly described in the previous section. Figure 4a,b shows the outer and inner geometric elements for which measurements were taken on models printed from the four materials in different print directions.

The following values, shown in Table 3, were recorded for the measurements of the outer geometric elements of the models printed from the four materials in different printing directions.

**Figure 5:**
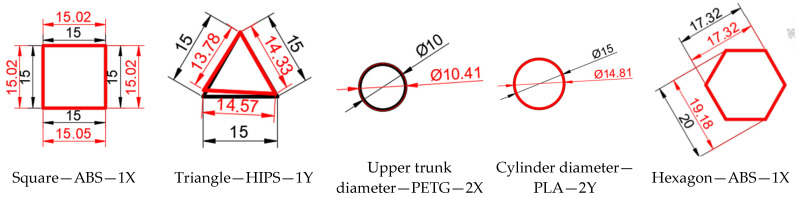
Representation of the dimensional differences in the external geometric elements between the design and the resulting parts that were printed using the additive FDM technology. Source: authors, based on research results.

The graph in Figure 5 was generated based on the data in Table 3 for just five elements made of different materials and with different orientations to the printing direction, in order to observe the dimensional differences in the external geometric elements resulting from parts printed using additive FDM technology.

Analysing the results presented in Table 3, we found that there are significant differences from a dimensional point of view in the external geometric elements of the parts made of ABS. Thus, the most deviations are in the square area printed on the 2X orientation, of 2.53%, the triangular area printed on the 1Y orientation, of 5.56%, the upper trunk diameter printed on the 1X orientation, of 4.8% and the distance between the diametrically opposite ends of the hexagon printed on 4.98% 1Y orientation.

Regarding the part obtained from HIPS, the most deviations are in the square area printed on the 2X orientation, of 5.93%, the triangular area printed on the 2X orientation, of 13.12%, the upper trunk diameter printed on the 2X orientation, of 5% and the distance between the ends of the diametrically opposed hexagons printed on the 2Y orientation, of 5.04%.

Regarding the part obtained from PETG, the most deviations are in the triangular area printed on the 1Y orientation, of 8.14%, the upper trunk diameter printed on the 1X orientation, of 5.4% and the cylinder top diameter printed on the 1Y orientation, of 3.09%.

Regarding the part obtained from PLA, the most deviations are in the triangular area printed on the 2Y orientation, of 6.15%, the upper trunk diameter printed on the 1X and 2X orientations, of 5.4% and the distance between the diametrically opposite ends of the hexagon printed on the 1Y orientation, of 3.19%.

It should be noted that certain shapes exhibit dimensions that are not substantially different from those defined during part design. This includes the square side and the distance between parallel sides of the hexagon in the X1 direction for ABS material. However, these cases are limited in number when considering the overall complexity of the part under analysis.

As the comparative analysis of the results presented in Table 3 shows, there are significant dimensional differences in the outer surfaces produced by the 3D printing process, both in terms of the printing direction for each of the four types of materials under analysis and in terms of significant dimensional differences in the same printing direction but for different materials. In summary, it has been determined that, in addition to the printer setting parameters, the printing direction and the type of material selected for 3D printing are factors that influence the dimensional accuracy of the parts produced.

The following values, shown in Table 4 were recorded for the measurements of the inner geometric elements of the models printed from the four materials in different printing directions.

**Figure 6:**
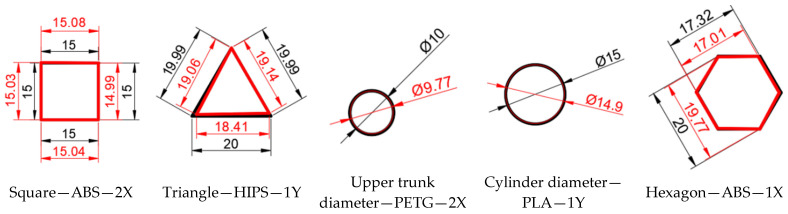
Representation of the dimensional differences in the internal geometric elements between the design and the resulting parts that are printed using the additive FDM technology. Source: authors, based on research results.

The graph in Figure 6 was generated based on the data in Table 4 for just five elements made of different materials and with different orientations to the printing direction, in order to observe the dimensional differences in the external geometric elements resulting from parts printed using additive FDM technology.

Analysing the results presented in Table 4, was find that there are significant differences from a dimensional point of view in the internal geometric elements of the parts made of ABS. Thus, the most deviations are in the triangular area printed on the 2X orientation, of 8.34%, the upper trunk diameter printed on the 2X orientation, of 3.2% and the distance between the diametrically opposite ends of the hexagon printed on the 2X orientation, of 3.52%.

Regarding the part obtained from HIPS, the most deviations are in the triangular area printed on the 1X orientation on all three sides, respectively, 12.36%—L1; 9.29%—L2; 6.32%—L3.

Regarding the part obtained from PETG, the most deviations are in the triangular area printed on the 1X orientation, of 6.55% and the cylinder diameter printed on the 1X orientation, of 5.48%.

Regarding the part obtained from PLA, the biggest deviations are in the triangular area printed on the 2X orientation, of 6.78% and the distance between the diametrically opposite ends of the hexagon printed on the 2X orientation, of 3.89%.

A comparison of the results in Table 4 reveals significant dimensional and interior surface differences in the 3D-printed exterior surfaces, both in terms of printing direction for each of the four types of materials and significant dimensional differences in the same printing direction but for different materials. In summary, the analysis indicates that, for interior surfaces, the dimensional accuracy of 3D-printed parts is influenced by printer setting parameters, printing direction and material type.

In light of these findings, we conclude that the primary objective of this study, as suggested by its title, has been successfully met. Our subsequent investigations will focus on the potential for enhancing the design of 3D-FDM manufactured parts through dimensional optimisation.

Starting from the hypothesis that the authors had in mind, an overview of the measured dimensional differences in the geometric elements in the FDM additive-printed parts of the four material types compared to those established at the design stage was obtained from the analysis of the data presented in tabular and graphical form.

These differences provided the proportions by which the dimensions had to be increased or reduced in the design phase to obtain a part with the desired actual dimensions. This technological aspect must be handled with care, as these dimensional differences can produce nonconforming parts (technological rejects).

As well as analysing the percentage changes in the dimensions of the FDM additive-printed parts, a gravimetric analysis of the 16 models was also undertaken to show how these dimensional changes affected the mass of the specimens. The gravimetric analysis was carried out using a Kern ABS 220–4N analytical balance, externally calibrated with a maximum weighing range of 220 g.

**Figure 7:**
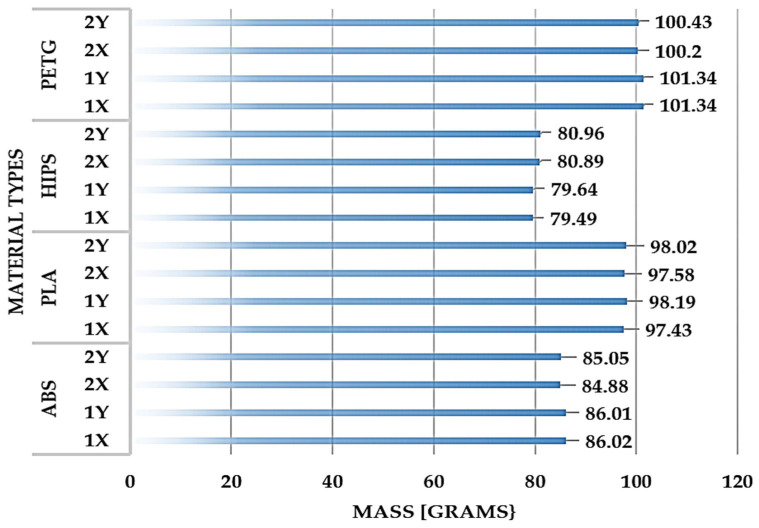
Gravimetric comparison of the parts made from the four materials using the additive technology of FDM. Source: authors, based on research results.

The results of the gravimetric analysis are summarised in Figure 7. The deviations from the masses determined by the 3D software are explained in the following paragraphs.

The gravimetric comparison of the parts made from the four materials using the FDM additive technology has produced the results which are presented below.

### 4.1. For Parts Printed from ABS Polymer

For ABS parts, the standard weight, determined using the specific density of ABS in a 3D environment, is 90.03 g.

Actual masses were obtained by weighing parts made with FDM additive technology using an ABS polymer in the print direction:

- 1X is 86.02 g.
- 1Y is 86.01 g.
- 2X is 84.88 g.
- 2Y is 85.05 g.

### 4.2. For Parts Printed from HIPS Polymer

For HIPS parts, the standard weight, determined using the specific density of ABS in a 3D environment, is 86.75 g.

Actual masses were obtained by weighing parts made with FDM additive technology using a HIPS polymer in the print direction:

- 1X is 79.49 g.
- 1Y is 79.64 g.
- 2X is 80.89 g.
- 2Y is 80.96 g.

### 4.3. For Parts Printed from PLA Polymer

For PLA parts, the standard weight, determined using the specific density of ABS in a 3D environment, is 105.65 g.

Actual masses were obtained by weighing parts made with FDM additive technology using a PLA polymer in the print direction:

- 1X is 97.43 g.
- 1Y is 98.19 g.
- 2X is 97.58 g.
- 2Y is 98.02 g.

### 4.4. For Parts Printed from PETG Polymer

For PETG parts, the standard weight, determined using the specific density of ABS in a 3D environment, is 104.60 g.

Actual masses were obtained by weighing parts made with FDM additive technology using a PETG polymer in the print direction:

- 1X is 101.34 g.
- 1Y is 101.34 g.
- 2X is 100.20 g.
- 2Y is 100.43 g.

Differences in weight between the values obtained and the normal weight are easy to observe and can be caused by several factors, such as the following:

- Experimental errors: When measuring and testing, there is always some experimental error that can affect the results obtained. This may be due to variations in the manufacturing process, to variations in the equipment used, or to other experimental variables that are not under control.
- Manufacturing variations: The materials used in the weight tests may be subject to manufacturing tolerances and allowable variations. Variations in the weight obtained may result from these manufacturing variations.
- Limitations of the equipment: The equipment used for the test or measurement may have inherent limitations or errors in it. These may have an influence on the results and contribute to the observed weight variations.

All dimensions of the parts to be produced by the FDM additive technology process must be corrected, in accordance with Table 3 and Table 4, so that the dimensions are those desired at the design stage and found on the production drawings and the weight is normal. Keeping to the technological parameters is important, as wastage will vary depending on the material used.

To conclude, maintaining the calculated geometric deviations is very important in the manufacturing process. If these deviations are not respected, the result will be a deformed or non-conforming part. This part will have to be removed and considered as scrap.

**Figure 8:**
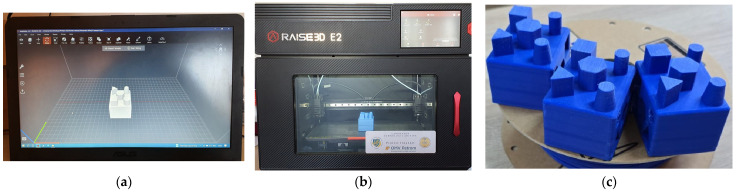
Model pieces made to confirm the results of the dimensional study. (**a**) Orientation of the parts on the table of the 3D printing machine according to the direction of printing; (**b**) production of the 2Y-orientated HIPS polymer parts of the Raise 3D E2 printer; (**c**) HIPS polymer parts resulting from 3D printing by FDM technology on 2Y printing direction. Source: authors, based on research stage.

In order to confirm the results of the study on the influence of materials and printing direction on the reliability of the dimensional values of the measured elements in relation to those desired at the design stage, we randomly selected and produced three pieces of HIPS polymer, oriented on the 2Y printing direction, modifying all the dimensional values by the percentages identified in the analysis of the measurement data to show the achievement of the dimensional accuracy desired at the design stage. This part is shown in Figure 8.

Measurements were performed using the same instrument and methodology described above, under laboratory conditions with the same ambient temperature values.

The dimensional values of the designed geometric elements were modified, with the specific percentages of each dimension, by additive printing using FDM technology on a randomly selected part, a HIPS polymer part with a 2Y printing direction. The values of the external and internal dimensions are given in Table 5 and Table 6, respectively.

All the items have the approximate values proposed in the design phase leading to the validation of this dimensional study, as can be seen from the values presented in Table 5. A simple observation is that some of the values exceed the desired value for which they should be declared non-compliant but considering that they are higher deviations and the dimensions are outside, they are recoverable rejects, because the adjustments can be made using conventional technologies. Of course, adjustments can be made to the percentage of variation in dimensional values at the design stage by refining the results by increasing the number of samples and measurements.

As can be seen from the values shown in Table 6, it is the same as for the external dimensions. The internal dimensions are approximately the same as those proposed during the design phase, which led to the validation of this dimensional study. A simple observation is that some of the values are lower than the desired values, for which they should also be declared non-compliant, but since they are smaller deviations and the dimensions are internal, they are recoverable rejects, since adjustments can be made using conventional technologies. Also, the percentage of variation in the dimensional values can be adjusted at the design stage by refining the results by increasing the number of samples and measurements.

Additionally, the mass values of the manufactured components post-dimensional correction are closely aligned with the estimates derived from the software utilised during the design phase, where the three-dimensional model was created, and its volume was assigned the specific polymer density.

## 5. Discussion

The present study focuses on measuring the dimensions of additive-printed 3D models made from different polymers (ABS, PLA, HIPS, PETG) using additive FDM technology. The polymers that were used, the 3D printer (Raise 3D E2) and the measuring equipment (Axiom Too HS CMM) that were used in the study are also presented.

Broadly speaking, the first part of the article presents the fundamentals of the dimensional study of 3D printing parts. This introductory part prepares the ground by presenting the polymers, equipment and methodology used to produce the parts to be studied. These will be studied and analysed to understand how the dimensional accuracy and variation of 3D-printed objects are affected by the polymers used and the orientation of the parts with respect to the printing direction.

- Printing challenges and material properties: A discussion of the properties of the materials is essential, as it highlights the unique characteristics of each of the polymers used. ABS, which is known for its strength and durability, presents challenges in terms of expansion and contraction during the printing process. PLA, a biodegradable material, has excellent properties for surface finishing, but is also faced with issues related to dimensional changes during the additive printing process. HIPS is an economical plastic that is easy to thermoform and has been observed to be the cheapest material for 3D printing, while PETG offers a balance between the properties of PLA and ABS. To accurately size 3D-printed materials, it is important to understand the properties of these materials.
- Factors that can influence dimensional variation: Factors that can cause dimensional variations in 3D-printed parts were also presented in this study. These factors include the thermal expansion of the material, errors in the calibration of the printer, cooling time, mechanical stress, environmental conditions and the accuracy of the design. Valuable information on potential sources of geometric element size variation is provided by discussing these factors.
- Sample production: The article also explains in detail how the 16 samples were produced, 4 for each polymer, with different print orientations. The need for consistency in the study is emphasised by mentioning the importance of following the printing parameters recommended by the manufacturers of the polymers used.
- Coordinate measuring machine: To achieve high confidence results in the study, a high-precision Axiom Too HS CMM was used. For high accuracy, the use of the PH10T touch probe and the RTP20 magnetic probe was chosen.
- Methodology used: The methodology is explicitly presented in the article, starting with the design of the CAD model and the selection of materials, and then continuing with the generation of the GCODE, the production of the samples, the measurements and the data that was recorded. This methodology provides a clear road map for the process of the research.

The second part of the study presents the aspects related to the analysis of dimensional differences in additive-printed parts using FDM technology. The parts were made from four different types of polymers (ABS, HIPS, PETG and PLA). The study compares the actual dimensions of the printed parts, as measured, with the dimensions defined at the design stage. The purpose of this analysis is to determine the percentage by which the dimensions need to be adjusted at the design stage to achieve the desired final dimensions and to avoid the production of non-conforming parts.

Purpose of dimensional analysis: In this article, an analysis will be carried out to understand the dimensional differences between the printed parts and their design specifications. To ensure that the final parts meet the desired dimensional requirements, these differences are essential.

Presentation of the data: The data resulting from the measurements required for the analysis are presented in both a tabular and a graphical form. The measured values have been plotted to two decimal places to make it easier to see the influence. The study carried out underlines the importance of carefully managing dimensional differences. These can lead to non-conforming parts or technological rejects. Accurate dimensions are essential for the functionality and quality of the final product in manufacturing and engineering.

In short, the aim of dimensional analysis is to provide valuable information on how the use of different types of polymer and different orientations on the print directions affect the dimensions of FDM additive-printed parts in comparison to the specifications given at the design stage.

The study also underscores the importance of accounting for dimensional differences, which can be corrected by adjusting printing quotas according to the percentage deviations identified.

This information is important for engineers and manufacturers to make the necessary adjustments during the design phase to ensure that the final parts meet the intended dimensions and quality standards.

Also, a gravimetric analysis of the parts produced by FDM additive technology from the four types of plastic (ABS, HIPS, PLA and PETG) has been undertaken to highlight how the dimensions obtained affect the mass of the parts. The gravimetric comparison of parts made from the four different polymer types using additive FDM is summarised graphically.

The analysis is carried out for each individual polymer and provides both the standard masses, calculated from their specific densities and volumes identified using a 3D medium, and the actual measured masses of the parts produced using the FDM technology, indicating the deviations from the standard weight.

The discrepancies between the predicted and actual weights of these parts are explained, and the potential factors contributing to these differences are identified. To avoid waste and produce compliant parts, the text emphasises the importance of maintaining the correct dimensions and technology parameters during the manufacturing process. Weighing the model part produced using additive FDM technology in HIPS polymer in the 2Y print direction, whose dimensions had been changed at the design stage, also confirms that this method of correcting part dimensions at the design stage is correct, an aspect confirmed by the insignificant differences between the actual weight and that estimated by the 3D software.

## 6. Conclusions

From the research performed by the authors, some conclusions can be drawn. First of all, this study provides a comprehensive analysis of the dimensional accuracy and variation in additive-printed 3D models made from different polymers (ABS, PLA, HIPS and PETG) using FDM technology.

This research highlights how important it is to understand each polymer’s unique properties when 3D printed.

Various factors, such as material thermal expansion, printer calibration errors, cooling time, mechanical stress, environmental conditions and design accuracy, that affect the dimensional accuracy of 3D-printed parts are discussed. This information will help to identify potential sources of dimensional variation.

Detailed information is given on how 16 samples, 4 for each polymer, were produced using different printed orientations. The importance of consistency in following recommended print parameters is emphasised.

Analysing the dimensional differences between printed parts and their design specifications is the main objective of the study. To ensure that the final parts meet the desired dimensional requirements, this analysis is critical. The measured data will be presented in both tabular and graphical form, making it easy to visualise the percentage changes in dimension for each polymer and for each orientation of the print. This helps to identify the necessary adjustments required to meet the design specifications.

To avoid non-conforming parts or technological waste, the study emphasises the importance of managing dimensional variation. Dimensional accuracy is very important for the product functionality and quality, and this can be improved by minimising dimensional errors by taking into account the percentage deviations of the printed model from the designed model and re-initialising the process after adjusting for them.

The study was extended with analysis that includes the mass of parts produced using the FDM additive technology from a range of different types of polymers. By comparing the actual measured masses with the standard masses calculated from densities and volumes, the relationship between dimensions and mass is highlighted. It verifies the effectiveness of correcting part dimensions during design by comparing the actual and estimated weights. This approach helps to eliminate waste and ensures that parts are produced to specification.

To summarise, this research provides valuable insights into how polymer selection, percentage ratio changes identified by practical methods and printing orientation affect the dimensional accuracy of 3D-printed parts. Engineers and manufacturers can use these findings to make informed adjustments to their designs, ultimately ensuring that the final parts will meet their intended dimensions and quality standards. The study also highlights the importance of maintaining correct dimensions and technology parameters during manufacturing to minimise waste and produce compliant parts.

## Figures and Tables

**Table 1:** Technical parameters of the materials. Source: authors, based on materials properties.

Material	ABS	PLA	HIPS	PETG
Density	1.04–1.12 g/cm^3^	1.24–1.44 g/cm^3^	1.03–1.07 g/cm^3^	1.27–1.31 g/cm^3^
Tensile strength	40–60 MPa	37–73 MPa	22–36 MPa	50–75 MPa
Young’s modulus	1.9–3.5 GPa	1.9–3.5 GPa	1.9–3.1 GPa	2–3 GPa
Impact strength	High	Poor	Very good	Very good
UV resistance	Satisfactory	Poor	Poor	Very good
Dimensional stability	Good	Good	Excellent	Excellent
Extrusion temperature	210–250 °C	160–220 °C	175–220 °C	245–255 °C
Chemical resistance	Resistance to several chemicals	Limited resistance to chemicals	Resistant to water and many common chemicals, but sensitive to organic solvents	Resistant to most common chemicals
3D printer compatibility	Good	Very good	Very good	Very good

**Table 2:** The 16 probes, 100%-filled specimens printed in different orientations. Source: authors, based on research stage.

	Direction	1X	1Y	2X	2Y
Polymer Type					
ABS		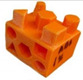	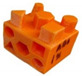	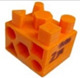	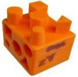
PLA		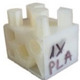	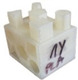	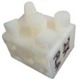	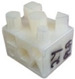
HIPS		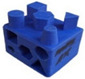	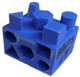	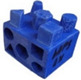	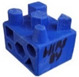
PETG		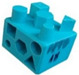	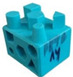	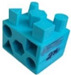	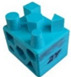

**Table 3:** External dimensions of the geometric elements were measured for the sixteen samples. Source: authors, based on measurements results.

Measured Geometrical Dimension	ABS				HIPS				PETG				PLA			
	1X	1Y	2X	2Y	1X	1Y	2X	2Y	1X	1Y	2X	2Y	1X	1Y	2X	2Y
Square side L1	15.02	14.69	15.34	15.02	15.23	15.23	14.16	14.57	15.12	15.28	15.32	15.20	15.24	15.21	15.18	15.31
Square side L2	15.02	15.07	15.03	15.04	14.88	14.95	15.02	15.03	14.93	14.90	14.86	14.83	14.91	14.87	14.85	14.86
Square side L3	15.05	14.73	15.38	15.32	15.19	15.31	14.48	14.73	15.04	14.93	15.15	15.28	15.23	15.24	15.30	15.36
Square side L4	15.08	15.07	15.02	15.03	14.91	14.93	14.99	14.95	14.90	14.83	14.83	14.97	14.96	14.91	15.04	14.95
Triangle side L1	14.93	14.58	15.15	14.89	13.74	14.33	13.58	13.62	14.58	13.87	14.42	14.42	14.45	14.96	14.54	14.66
Triangle side L2	14.74	14.32	15.08	14.71	13.65	14.57	14.07	14.05	14.33	13.91	14.14	14.19	14.72	14.66	13.95	14.13
Triangle side L3	14.82	14.21	15.18	14.74	13.79	13.78	13.26	13.32	14.19	13.93	13.97	14.27	14.54	14.27	14.34	14.36
Upper trunk diameter	10.46	10.48	10.48	10.47	10.39	10.36	10.50	10.42	10.54	10.21	10.16	10.41	10.54	10.00	10.54	10.41
Cylinder top diameter	15.10	15.09	14.91	15.04	15.01	14.90	14.86	14.92	14.78	14.55	14.76	14.74	14.77	14.57	14.83	14.81
Distance between the parallel sides of the hexagon	17.32	17.36	17.27	17.20	17.19	17.46	17.27	17.28	17.17	17.20	17.11	17.15	17.19	17.21	17.24	17.24
Distance between the diametrically opposite ends of the hexagon	19.18	19.05	19.19	19.27	19.24	19.35	19.26	19.04	19.74	19.88	19.58	19.75	19.41	19.38	19.70	19.69

**Table 4:** Internal dimensions of the geometric elements were measured for the sixteen samples. Source: authors, based on measurements results.

Measured Geometrical Dimension	ABS				HIPS					PETG			PLA			
	1X	1Y	2X	2Y	1X	1Y	2X	2Y	1X	1Y	2X	2Y	1X	1Y	2X	2Y
Square side L1	14.90	14.90	15.08	14.94	15.03	15.11	14.95	14.81	15.13	14.05	15.05	15.12	15.01	15.00	15.04	14.93
Square side L2	14.84	14.90	14.99	14.92	15.23	15.14	14.86	15.00	15.01	15.06	14.86	14.95	14.88	14.79	14.88	14.94
Square side L3	14.86	14.88	15.04	14.96	15.09	15.08	14.92	14.95	15.15	14.98	14.98	15.06	15.08	14.99	14.88	14.93
Square side L4	14.81	14.91	15.03	15.26	15.33	15.29	14.91	14.96	14.97	14.98	14.97	15.00	15.08	14.82	14.96	14.94
Triangle side L1	18.80	18.96	18.74	19.09	17.80	19.14	19.15	19.25	19.25	19.34	18.82	19.40	19.66	19.20	19.18	19.48
Triangle side L2	18.94	19.14	18.46	19.31	18.30	18.41	19.34	19.42	19.29	19.08	19.39	18.89	18.89	19.10	18.73	19.20
Triangle side L3	19.05	19.07	19.28	19.33	18.81	19.07	19.34	19.29	18.77	19.49	19.43	19.58	19.22	19.57	18.88	19.35
Upper trunk diameter	9.98	9.86	10.32	10.05	9.81	9.88	10.10	9.94	9.92	9.88	9.77	10.03	9.49	10.07	10.08	10.12
Cylinder diameter	14.79	14.80	14.88	14.82	14.85	14.91	14.90	14.89	14.22	14.51	14.94	14.51	15.05	14.90	14.93	15.00
Distance between the parallel sides of the hexagon	17.01	17.17	17.64	17.56	17.10	17.00	17.22	17.40	17.20	17.38	17.28	17.29	17.11	16.95	17.13	17.22
Distance between the diametrically opposite ends of the hexagon	19.77	19.79	19.32	19.82	19.90	19.79	19.79	19.77	19.54	19.70	19.86	20.05	19.70	19.84	19.25	19.88

**Table 5:** Outer dimensions of geometric elements for FDM-printed parts in HIPS polymer, 2Y print direction. Source: authors, based on measurements results.

External Dimensions		Square Side L1	Square Side L2	Square Side L3	Square Side L4	Triangle Side L1	Triangle Side L2	Triangle Side L3	Upper Trunk Diameter	Cylinder Diameter	Distance Between the Parallel Sides of the Hexagon	Distance Between the Diametrically Opposite Ends of the Hexagon
Material	Printing Direction	mm	mm	mm	mm	mm	mm	mm	mm	mm	mm	mm
HIPS_1_	2Y	15.02	15.01	15.01	15.00	15.01	15.00	15.01	10.01	15.00	17.33	20.01
HIPS_2_	2Y	15.01	15.00	15.00	15.02	15.01	15.00	15.00	10.00	15.01	17.34	20.00
HIPS_3_	2Y	15.02	15.02	15.01	15.02	15.01	15.01	15.00	10.01	15.00	17.32	20.00

**Table 6:** Inner dimensions of geometric elements for FDM-printed parts in a HIPS polymer, 2Y print direction. Source: authors, based on measurements results.

External Dimensions		Square Side L1	Square Side L2	Square Side L3	Square Side L4	Triangle Side L1	Triangle Side L2	Triangle Side L3	Upper Trunk Diameter	Cylinder Diameter	Distance Between the Parallel Sides of the Hexagon	Distance of the Diametrically Opposite Ends of the Hexagon
Material	Printing Direction	mm	mm	mm	mm	mm	mm	mm	mm	mm	mm	mm
HIPS_1_	2Y	14.99	15.00	14.97	15.00	14.99	15.00	14.99	10.00	15.00	17.30	20.00
HIPS_2_	2Y	14.98	15.00	14.97	14.99	15.00	14.99	14.99	10.00	14.99	17.32	20.00
HIPS_3_	2Y	15.00	15.00	14.99	14.99	14.99	15.00	14.99	10.00	15.00	17.30	19.99

## Data Availability

The original contributions presented in this study are included in the article. Further inquiries can be directed to the corresponding authors.
